# An Explorative Study to Assess the Neuropsychiatric Impact of COVID-19

**DOI:** 10.1192/bjo.2022.194

**Published:** 2022-06-20

**Authors:** Baidyanath Ghosh Dastidar

**Affiliations:** Calcutta National Medical College, Kolkata, India

## Abstract

**Aims:**

To assess the neurocognitive and psychiatric impact of SARS-CoV-2 in COVID-19 recovered patients in a district COVID hospital in West Bengal, India.

**Methods:**

A total of 300 COVID-19 recovered patients who had suffered from SARS-CoV-2 and admitted at a district COVID hospital in West Bengal were selected by simple random sampling method. Informed consent was obtained from these patients. Subsequently a questionnaire based interview was conducted by trained clinical psychologist. The following scales were administered BDI (Depression), BAI, HAM A(Anxiety), SF 36 (Quality of Life), SCL 90 (Psychopathology), Addenbrooks scale (Neuro Cognitive impairment), socio demographic proforma which included vaccination status, pulmonary involvement and medical interventions.

The data were analysed by SPPS and compared with matched control group and the following statistical tools were used - independent t test, spearman's rho, chi square test, linear regression analyses and z test.

**Results:**

The results of our study do not indicate any statistically significant differences in the psychosocial parameters (depression, anxiety, psychopathology and quality of life) between case and control group.

Neurocognitive deficits not statistically significant in study population.

Delirium experienced during admission process and vascular insult such as stroke significant in case versus control group.

**Conclusion:**

Our study indicates that COVID-19 does not have any significant psychological or neurocognitive impact.

Our study was one of the few interview based studies conducted on COVID recovered patients.

Certain studies collected data from emergency room case records / meta analysis to suggest that COVID-19 may have a psychological sequel in the long term.

Our study and similar interview based studies contradict this hypothesis.

